# A New Metric of Inclusive Fitness Predicts the Human Mortality Profile

**DOI:** 10.1371/journal.pone.0117019

**Published:** 2015-01-21

**Authors:** Saul J. Newman, Simon Easteal

**Affiliations:** John Curtin School of Medical Research, Australian National University, Canberra, Australia; University of Arkansas, UNITED STATES

## Abstract

Biological species have evolved characteristic patterns of age-specific mortality across their life spans. If these mortality profiles are shaped by natural selection they should reflect underlying variation in the fitness effect of mortality with age. Direct fitness models, however, do not accurately predict the mortality profiles of many species. For several species, including humans, mortality rates vary considerably before and after reproductive ages, during life-stages when no variation in direct fitness is possible. Variation in mortality rates at these ages may reflect indirect effects of natural selection acting through kin. To test this possibility we developed a new two-variable measure of inclusive fitness, which we term the extended genomic output or EGO. Using EGO, we estimate the inclusive fitness effect of mortality at different ages in a small hunter-gatherer population with a typical human mortality profile. EGO in this population predicts 90% of the variation in age-specific mortality. This result represents the first empirical measurement of inclusive fitness of a trait in any species. It shows that the pattern of human survival can largely be explained by variation in the inclusive fitness cost of mortality at different ages. More generally, our approach can be used to estimate the inclusive fitness of any trait or genotype from population data on birth dates and relatedness.

## Introduction

Patterns of mortality vary greatly among biological species [1]. In many species there is a general increase in the rate of mortality with age [1]. In some, however, mortality rate is constant throughout life (e.g., *Hydra magnipapillata* [1]). In others, the general trend of increasing mortality is reversed and mortality rate either declines during some life stages (e.g., the alpine swift *Apus melba* [1]) or continuously throughout life (e.g., the desert tortoise *Gohoerus agissizii* and the white mangrove *Avicennia marina* [1, 2]).

This variation presents a challenge for evolutionary theories of aging [3–5]. These theories were developed to explain the apparent paradox of aging—how natural selection, acting to maximize direct fitness, can cause mortality rates to increase across the life span. They are based on Fisher’s initial proposal that the Malthusian parameter (*m*), estimated by the Euler-Lotka equation (ELe) [6, 7], is a measure of the average individual fitness in a population (see Fisher [8]; p.26). In the biological application of Lotka’s continuous equation:

$$\int_{x}^{\infty }e^{-mx}f_{x}l_{x}dx=1,$$

*f*_x_ is the probability of reproduction at age *x*, and *l_x_* is the probability of survival to age *x*.

Euler proposed that survivorship *l* necessarily remains stable or declines with increasing age *x*, such that *l_x_ ≥ l_x+1_* [9]. Hamilton extended this argument, proving that the fitness *w* gained by survival to age *x* must decline with age under the ELe, such that *w_x_ ≤ w_x+1_*. The ELe therefore predicts that selection *must* favor increasing mortality rates with age in all populations [1, 10–12]. Hamilton’s theory provided a solution to the paradox of aging. However, as Hamilton detailed [10], this theory does not provide a complete explanation of the observed pattern of mortality.

Human populations provide an excellent context in which to develop and test new theoretical approaches to evolution of aging. Human longevity, and by extension, age-specific mortality rates, are one of the most extensively documented complex traits in any species. Billions of linked birth and death certificates exist across many human populations [13].

Humans, like some other species, have a mortality profile with two features that are not predicted by the ELe or related population matrix models. There is a rapid decrease in mortality rate from a peak in infancy to near-zero during adolescence, and a gradual increase of mortality rate well into post-reproductive life [10, 14]. No variation in direct fitness occurs during these non-reproductive life stages, as individuals have no reproductive output. Since this variation does not reflect direct fitness effects, it must be either non-adaptive, or it must reflect hidden effects of inclusive fitness on the mortality profile.

Pairwise kin-dependent fitness effects on survival have been observed in humans [15], especially in postmenopausal women [16, 17]. These studies indicate kin-specific inclusive fitness effects, but they do not measure the collective effect of kinship on mortality, or provide an explicit measure of inclusive fitness.

To investigate more fully the role of inclusive fitness in the evolution of mortality profiles, we developed a novel metric of inclusive fitness that incorporates interactive kinship effects. We define inclusive fitness (*w_inc_*) of an individual as the representation of that individual’s genome in a population over time. *w_inc_* can be direct, reflecting descent through reproductive success, and indirect, reflecting descent through the reproductive success of relatives. The inclusive fitness of a class of individuals with the same genotype or phenotype is the mean inclusive fitness of individuals in that class (${\bar{w}}_{inc}$). The relative inclusive fitness of a class of individuals is their mean inclusive fitness relative to the mean inclusive fitness of other individuals in the population.

To estimate *w_inc_* we use a measure of inclusive fitness that we term the extended genomic output or EGO. To obtain EGO, we divide an individual’s genome into a finite number (*p*) of discrete genomic elements of arbitrary size. Here we make the simplifying assumptions that all genomes are of equal size, and that the probability of two individuals sharing a genomic element is given by Wright’s [18] coefficient of relatedness *r.* Thus an individual shares *p*/2 genomic elements with first-degree relatives, *p*/4 elements with second-degree relatives, and so on.

EGO is defined for a specific period. For an *ith* individual, EGO during the period *t … t+x* is given by:

$$EGO_{i}=\sum_{t}^{t+x}r_{ij}$$

where *r_ij_* is the coefficient of relatedness of the *ith* individual to each *jth* individual born during *t…t+x*. The inclusive fitness of any trait is obtained by taking the mean EGO of all individuals sharing that trait.

The age of mortality of an individual may substantially affect the survival, resource availability and reproductive rate of their surviving relatives [12–14]. EGO captures these effects, measuring how much inclusive fitness accumulates at a fixed census time after birth (*t*), irrespective of whether or not an individual is still alive at that time.

Here we compare the predictive accuracy of ELe and EGO using the mortality profile of the Casiguran Agta. The Agta are an isolated hunter-gatherer population indigenous to Luzon, Philippines, with demographic characteristics approximating those of modern humans during much of their evolution [19]. This population has been monitored longitudinally for over 60 years for parent-child relationships, migration, and dates of birth and mortality [19].

## Methods

We calculated the mortality profile for the Agta population from data in the Agta Demographic Database [19], which contains raw demographic data of the Agta population, published with the full permission of the Agta people.

Using these data we estimated direct fitness costs of mortality across the life course following Hamilton [10], solving the ELe using Coale’s method [20] in the “Lotka” package [21] (S1 Materials). We also conducted an elasticity analysis to estimate the direct fitness effect of small perturbations in age-specific mortality rates at different ages, using matrix-based population models (*vis.* Caswell [22] and Jones [14]; S1 Materials) implemented in the “popbio” package in R [23].

We then measured the inclusive fitness cost associated with mortality at different ages using EGO. From the Agta population data we removed 17 individuals who failed basic quality controls (see S1 Materials for details), and indexed all remaining individuals in the Agta population with parents who were born and remained within the peninsular Agta community during the study.

Growth in the diameter and complexity of the Agta genealogy graph (S1 Fig.) inflated relatedness estimates over time, through the addition of progressively higher-degree relationships (S2 and S3 Figs.). To eliminate this source of bias, we measured EGO using strictly delimited genealogies. EGO was calculated using genealogy sub-graphs constructed from all direct descendants of an individual’s parents (parental EGO; n = 2,703), or all direct descendants of an individual’s grandparents (grandparental EGO; n = 1,725; S3 Fig.).

As inclusive fitness is a function of both direct reproduction and the reproduction of relatives, individuals accumulate inclusive fitness after they have died. EGO was therefore measured for all genealogies documented to *t* years after birth, regardless of whether the individual was still alive at time *t*. Within each sub-genealogy, we calculated EGO gained by an individual from the birth of their oldest ascendant relative, to some fixed time *t* after the individual’s birth, independent of survival (S4 Fig.).

The correlation coefficient between the EGO-predicted cost of mortality at each age, and the observed probability of death at that age, was calculated for each census time *t* with > 100 documented individuals. This process was repeated for all census times *t* = 0 to *t* = 83, using both parental and grandparental-delimited genealogies (S5 Fig.). The accuracy of each EGO prediction was then compared to an equivalent estimate for the ELe. Additional methodological details are provided in the S1 Materials.

The small sample size of the Agta demographic database potentially reduced the accuracy of our predictions for both the EGO and ELe models. We therefore applied the Agta-derived EGO predictions to mortality profiles from 59 populations with more complete demographic data based on United Nations data [7]. These Agta-specific EGO predictions were then compared to ELe-based predictions calculated from within these 59 populations (S1 Materials).

Finally, we tested whether fitness cost estimates obtained using EGO were robust to emigration of descendant relatives from the survey area by repeating our EGO analysis after removing all individuals with more than 2 relatives dying in unknown locations.

## Results

**Figure 1:**
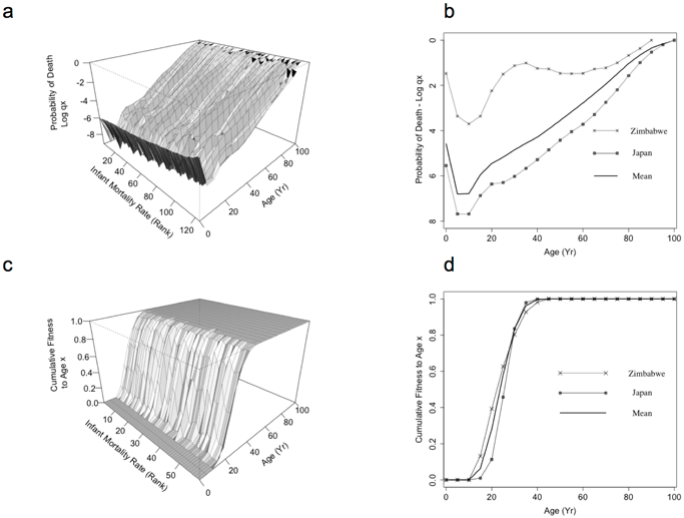
Variation in age-specific mortality and the accumulation of direct fitness. (a) Age-specific mortality profiles for populations in the most populous 132 nations (the UN Populations), ranked by infant mortality rate. (b) Range of human age-specific mortality profiles across countries with the lowest (Japan) and highest mortality rate (Zimbabwe; during a nation-wide cholera epidemic). (c) Accumulation of direct fitness with age, estimated using the Euler-Lotka equation for females in the 59 UN populations with available data. (d) Range of direct fitness accumulation across the life-course, measured by the ELe.

The mortality profile observed in both Agta and UN populations is characterised by relatively high mortality rates in infancy, which rapidly decline to a minimum at adolescence and remain low until the age of sexual maturity, after which mortality rises gradually with age (Fig. 1a). This distribution is robust to environmental variation. Paired mortality profiles of the 132 most populous nations (the UN Populations) [13] are, on average, highly correlated with each other (*r_mean_* = 0.95; *r*_min_ = 0.77; Fig. 1a, 1b), despite the modifying effects of substantial economic, social, medical, political and environmental differences.

**Figure 2:**
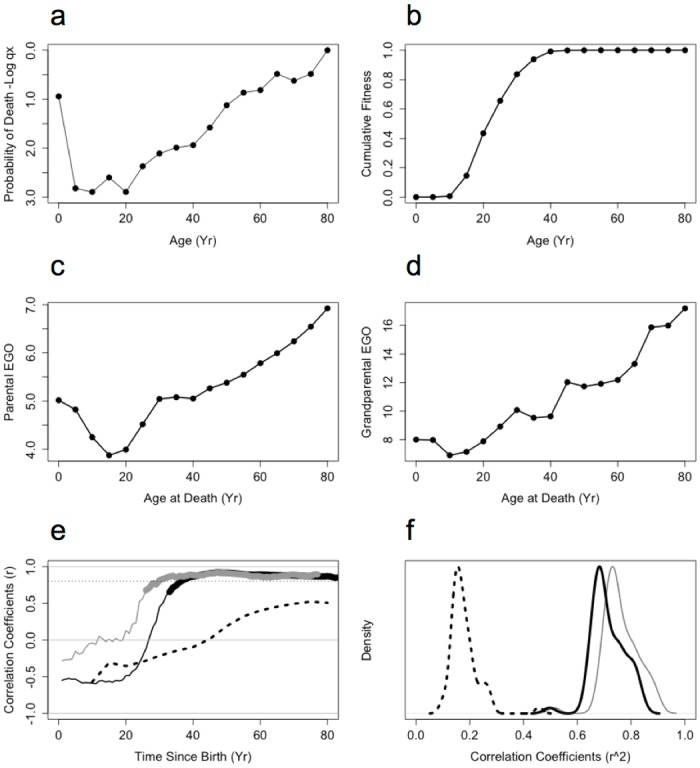
Comparison of Agta mortality profiles predicted by ELe and EGO. (a) Age-specific probability of mortality *qx*. (b) Mortality profile predicted by ELe. (c) Mortality profile predicted by parental EGO. (d) Mortality profile predicted by grandparental EGO. (e) Correlation coefficient between *qx* and ELe (dashed line), parental EGO (black line) and grandparental EGO (grey line) across a range of census times. Heavy lines indicate *p* <0.01. (f) Distributions of correlation coefficients between *qx* profiles of 59 UN populations and the ELe values calculated directly from these populations (dashed line), and Agta-derived parental (black solid line) and grandparental (grey line) EGO values.

Consistent with results for the UN populations (Fig. 1c–d), age-specific probability of mortality in the Agta (Fig. 2a) is only weakly correlated with the ELe-estimated fitness effects of mortality at different ages (*r* = 0.26; Fig. 2b). This correlation coefficient is unchanged when elasticity analysis is applied to the data (*r* = 0.26; S1 Materials).

EGO, measured at a census time of *t* = 45 years after birth, accurately predicts the age-specific probability of mortality across the Agta life span (*r* = 0.9; Fig. 2c–d). *t* = 45 was initially chosen as it is the time when variation in direct reproduction and the ELe should be maximally informative of mortality profiles.

The EGO prediction is robust to variation in census time. Both the ELe and EGO were poor predictors of the overall mortality profile for census times below *t* = 20 years (Fig. 2e), because there is limited inclusive and direct fitness accumulated during this period. However, the predictive value of EGO rapidly increases for all census dates above *t* >30 years (Parental EGO, *r*_mean_ = 0.65; Grandparental EGO, *r*_mean_ = 0.85, solid lines Fig. 2e). In contrast, there is only a marginal increase in the accuracy of the ELe predictions over this period (Fig. 2e, dotted line).

EGO obtained from the limited Agta dataset is also better able to predict the mortality profiles of 59 UN populations (*r_median_* = 0.63 and 0.66, for Parental and Grandparental EGO respectively) than the ELe, even though the ELe is calculated using fertility data directly observed in these populations (*r_median_* = 0.17, Fig. 2f).

## Discussion

Until now it has been difficult to assess the validity of inclusive fitness models, because they have lacked a clear metric. Using EGO we have shown that a simple inclusive fitness model provides an accurate prediction of a complex, demographically important life history trait that is not accurately predicted by direct fitness models. Our inclusive fitness model, constructed using only birth dates and relatedness coefficients, better predicts the evolution of the human mortality profile than current survivorship-dependent life history approaches.

Some residual variation in the mortality profile remains unexplained by our inclusive fitness model. Large sample sizes are required to accurately estimate mortality profiles [24], and it is possible that this residual variation reflects limited power of our analysis due to small sample size or the limited depth of genealogical information available in the Agta. We cannot, however, discount the possibility that some variation is non-adaptive and that it cannot be fully accounted for by an inclusive fitness model.

Our results add to existing evidence for the inclusive fitness benefits of post-reproductive survival in humans [15, 17], which may also explain post-reproductive survival in other species [25]. EGO also predicts that mortality incurs a lower inclusive fitness cost during infancy than during childhood, which provides an explanation for relatively higher mortality rates observed in infants than in children.

According to the “mutation accumulation” hypothesis [5], deleterious mutations climb local selective gradients toward ages where they incur the lowest fitness cost. Mortality is thus concentrated in infancy by a local selective gradient driving the progressively earlier expression of deleterious mutations. The result is a 31-fold higher rate of fatal genetic disorders in infants compared with children [26].

There are practical constraints to our application of EGO to the Agta population. In particular, the size of the study population required use of delimited genealogies. We also used a simple model that did not account for genealogical errors or for variance in the proportion of the genome transmitted between generations.

However, these constraints are not fundamental limitations. EGO is a general metric of inclusive fitness that can be applied to any trait or genotype and which can be measured directly, without pedigrees or inheritance models, using population-level genotype data. While the ELe treats individuals as isolated units, EGO deliberately captures complex interactions between individuals.

Direct fitness models are widely used in evolutionary biology as the basic metric of natural selection [27], and are fundamental in the construction of demographic models [28]. We have demonstrated that a simple inclusive fitness model better explains the evolution of the human mortality profile, a trait of central importance in demography. More broadly, this general measure of inclusive fitness opens up more than 50 years of contrasting hypotheses on inclusive and direct fitness to empirical testing.

## Supporting Information

S1 Materials Extended methods. (DOCX)

S1 Fig Kinship complexity within a small human population (N = 4242). Each node is one individual, connected to its offspring by a directed edge. The population is dominated by a single kinship network (a, n = 3530), with numerous smaller unconnected kinship graphs (b, n = 266), and unrelated individuals (c, n = 532).
(TIFF)

S2 Fig Increasingly complex patterns of biological relationship resulting from the accumulation of higher-degree relationships over time. (a) Network diagram showing the diversity of paths (grey edges) connecting a “father-daughter” pair. Traditional relationships such as “father-daughter” capture information from a single genealogical path (dashed line), whereas many higher-order connections (i.e path shown in black) exist. These higher-order relationships affect coefficients of relatedness in complex patterns: for father-daughter pairs (b) deviations from the expected *r* = 0.5 value are relatively minor, but for higher-order relationships such as “aunt-niece” and “uncle-nephew” categorical and observed *r* values deviate unpredictably in complexity and magnitude (c).
(TIFF)

S3 Fig The use of constrained genealogical subsets to eliminate bias in coefficients of relatedness *r* arising because of variation in the completeness of genealogies. (a) The uncorrected values of *r* were associated with a clear bias in relatedness estimates, as individuals entering the genealogy later were on average more related to the population. The use of either parent-delimited (b) or grandparent-delimited (c) genealogies eliminated this bias by excluding higher-degree relationships from the calculation of coefficients of relatedness *r*. Smoothed values shown as variable black line, black horizontal line shows population mean coefficient of relatedness.
(TIFF)

S4 Fig The selection of constrained genealogical subsets for estimating inclusive fitness. (a) A pattern-matching algorithm found every *ith* individual in the population with a full set of either parents or grandparents. (b) The direct descendants of all individuals in the population matching these criteria were collected (delimited by dashed line in b-c), creating a delimited sub-graph of the original genealogy. (c) We calculated the coefficient of relatedness *r* between the *ith* individual and all of their *jth* relatives within this sub-graph, and time-sorted the data relative to the *ith* individual’s date of birth. (d) We then measured the sum of *r* between each *ith* individual and their *jth* relatives across all periods from *t* = 0 to *tx*, where *t* = 0 is the earliest birth in the sub-graph and *tx* ranges from the birth of the *ith* individual to the last population census. This gave us our EGO measure of inclusive fitness over time.
(TIFF)

S5 Fig The relationship between the EGO-predicted probability of mortality and the observed probability of mortality across increasing census ages in the Agta population. At birth, EGO is not significantly correlated with the age-specific probability of mortality (*p*>0.01). As time since birth increases to *t* > 30, both parental (a) and grandparental EGO (b) begin to closely approximate the age-specific probability of mortality (*p* < 0.01; *r* > 0.8), despite considerable reductions in sample size.
(TIFF)
