# Supplementary material for: A New Metric of Inclusive Fitness Predicts the Human Mortality Profile
Source: PLoS One. 2015 Jan 21;10(1):e0117019. doi: 10.1371/journal.pone.0117019 (PMC4301870; doi:10.1371/journal.pone.0117019)
Supplement: S1 Materials — (DOCX) [file pone.0117019.s001.docx]

**Supporting Information:**

**Extended Methods**

**Analysis of the Agta Demographic Database**

The Agta Demographic Database [1] consists of the records of 4,300 Agta individuals, published with the full consent of the Agta community. We downloaded Version 2.0, 2011 or the database on 7 Mar 2013. Agta IDs were recoded as sequential integers to enable rapid indexing. We removed 11 individuals from the database who were pre-term infants at the time of the last population census (IDs 20046, 20053, 20056, 20058, 20062:20068). We removed six individuals who were encoded as the parents of recorded individuals (IDs 7587, 7826, 7922, 8076, 6082, 7827), but who had not been assigned their own unique database entries. We also removed individuals who had emigrated during the course of the study (n = 147), or who had lived in a non-surveyed area during the study (n = 102).

We represented the Agta genealogy as directed acyclic graphs (DAGs), with individuals added in order of birth date and represented as nodes connected to their offspring by directed edges (Fig. S1). As individuals are added new DAGs are formed, existing DAGs accumulate more nodes, and DAGs coalesce to form large connected sub-graphs.

As DAG size increases, higher-degree relationships between individuals increasingly contribute to the estimate of *r* (Fig. S2a). Late inclusion in the Agta DAG was associated with higher mean coefficients of relatedness to the population (Fig. S3a), due to the more complete inclusion of higher-degree relationships in the population sample, biasing estimates of inclusive fitness.

The use of kinship classifications with a fixed coefficient of relatedness, such as “father” or “second cousin”, was tested as a solution for addressing the time-dependent increases in *r* observed in the DAG. However, use of kinship classifiers omitted much of the complexity of relationships within the DAG, effectively measuring only one of the many biological paths connecting related individuals (Fig. S2a) and underestimating the variance in coefficients of relatedness (Fig. S2b, S2c).

**The measurement of EGO**

To eliminate bias due to the increase in *r* associated with genealogical completeness, we restricted our measurement of inclusive fitness to EGO gained through a restricted subset of relatives. We also excluded individuals with incompletely documented genealogies, by removing any individuals with a parent from a non-surveyed area (n=102) or whose parents had emigrated out of the survey population during the course of the study (n=147).

All remaining individuals with a fully documented set of parents (n=2301) or grandparents (n= 1308) were found via a pattern-matching algorithm within the larger DAG (Fig. S4a and S4b). This produced “grandparental” or “parental” ascendant DAGs containing all ascendants to either 1 or 2 degrees of relatedness.

We then collected all individuals directly descended from members of these “parental” and “grandparental” ascendant DAGs (dashed box, Fig. S4b and S4c) and incorporated them into the delimited parental and grandparental DAGs. This produced a large number of directly comparable, time-sorted genealogies. Each of these delimited genealogies included an *ith* individual, all of their *n*-degree ascendants (either parents, or parents and grandparents), and all individuals directly descended from the parents or grandparents of the *ith* individual.

We measured coefficients of relatedness between the *ith* individual and all other individuals within each parental- and grandparental-delimited DAG. This captured variation in *r* arising from a controlled subset of relationships.

Coefficients of relatedness within these DAGs were independent of the date at which an individual entered the population sample, eliminating the previously observed time-dependent change in *r* (Fig. S3).

Using these within-genealogy coefficients of relatedness, we measured the EGO of all *ith* individuals possessing a parental or grandparental DAG (Fig. S4d).

EGO measures the inclusive fitness of an individual across a time period *t…t+x*. We measured EGO from the earliest birth of all *n*-degree ascendants to a census time *tx*, where *tx* is measured relative to the birth date of the *ith* individual (*t0*). This measure of EGO includes all individuals with observable genealogies over the census period, regardless of their observed age at death. Thus, for example, inclusive fitness associated with death at age 4 can continue to accumulate for *t* > 4 years due to subsequent the reproductive success of relatives. EGO is therefore measured for individuals who are both living and deceased at each census time.

We initially measured EGO to a census time of *tx* = 45 years (Fig. 2c, d), when the ELe is maximally informative, using both the “parental” and “grandparental” delimited DAGs.

The relationship between each individual’s EGO at *tx* = 45, and their observed age at death, was modeled using a nonparametric nonlinear regression based on generalised product kernels [2]. This nonparametric regression was used to predict the EGO associated with mortality at 5-yearly intervals, and these predicted values compared by linear regression with the observed age-specific probability of mortality *qx* (*r* =0.90 and *r* =0.91; parental and grandparental EGO respectively). To ensure that our approach was not sensitive to variation in census time, we repeated this approach for each census time with >100 documented individuals (*tx* = 0 to *tx* = 83; Fig. S5). Variation in *tx* > 30 had very little effect on the correlation between EGO and qx.

Bias in EGO predictions may have been introduced by emigration of relatives out of the surveyed area during the course of the study. We estimated emigration bias by counting the total number of relatives dying in an unknown location. This estimate was approximate, as it included both individuals who had simply died in an unknown location within the survey area, and individuals who had died at an unknown location after emigrating.

All individuals with >2 relatives that had died in an unknown area were then excluded from analysis, and an estimate of the Agta mortality profile was recalculated for both parental and grandparental EGO. Despite a far smaller sample size, both parental and grandparental EGO still predicted a high degree of the variation in mortality profiles (*r* = 0.64 and *r* = 0.81 respectively; *t* = 45).

**Quantifying the Age-specific fitness cost of mortality using the ELe in the Agta and United Nations Populations.**

We assessed the relative performance of the ELe in predicting the age-specific probability of mortality using population mortality and fertility data from the 2011 World Population Prospects report [3]. The World Population Prospects report provides age-specific fertility and mortality data, and where possible mortality data is decomposed into both age and cause into the ~1400 categorical causes of death outlined in the WHO international cause of death codes.

For each country, mortality and fertility data were obtained for the most recent available census. Countries with fewer than 1,000 deaths per annum were omitted from analysis to remove sampling noise introduced by limited sample sizes.

To assess the similarity of age-specific mortality profiles across the UN countries, we estimated the correlation coefficient between age-specific mortality profiles for all pairs of these 132 countries using a linear model. Correlations between each pair of countries measured the similarity of the shape of mortality profiles, independent of shifts in the absolute magnitude of overall population mortality profile. The distribution of the resulting correlation coefficients is presented in Figure 1 of the main text.

Of the UN countries, 59 had contemporary measures of age-specific fecundity within the UN database. Using the “Lotka” package [4], we used these age-specific measures of mortality and fecundity to measure the fitness cost of mortality at each age.

For every UN population with fecundity data, and for the Agta population, we estimated the direct fitness gained after each age by the female population using the ELe, solved using Coale’s method [5]. This value measured the total fraction of fitness gained at a given age. The inverse of this value provided the mean fraction of direct fitness lost by mortality at each age.

We initially applied the ELe to predict the female-only mortality profile in the Agta, for which the ELe predicted only a small degree of the variation (*r* = 0.08). This is a more technically correct approach, as the ELe is specifically a one-sex demographic model [6] and cannot accurately integrate information from both sexes [7].

However, we considered that the observed correlation coefficient reflected the limited size of the female Agta population, and constituted an uneven comparison with the EGO based prediction. Male and female mortality profiles are highly correlated in shape within human populations, despite generally higher male mortality rates. We therefore measured the correlation between the ELe measurements and the combined Agta mortality profile from both sexes (*r* = 0.26).

The ELe is often implemented in matrix population models. Matrix models, in particular elasticity analysis, are useful in simulating the effect of small instantaneous changes in the mortality profile on direct fitness. We therefore conducted an elasticity analysis of the Agta population using the “popbio” package in R [8], which yielded an almost identical prediction to our previous ELe-based analysis (*r* = 0.26).

**References**

1. Headland J, Headland T, Uehara R (2011) Agta Demographic Database: chronicle of a hunter-gatherer community in transition. Available: http://www.sil.org/resources/publications/entry/9299. Accessed 10 December 2012.

2. Hayfield T, Racine JS (2008) Nonparametric Econometrics : The np Package. J Stat Softw 27: 1–32. doi:10.1198/jasa.2001.s374.

3. United Nations, Department of Economic and Social Affairs, Population Division. World Population Prospects: The 2010 Revision, CD-ROM Edition. (2011).

4. Riffe T (2013) Lotka. Available: https://sites.google.com/site/timriffepersonal/r-code/packagedownloads.

5. Coale AJ (1957) A New Method for Calculating Lotka’s r-the Intrinsic Rate of Growth in a Stable Population. Popul Stud (NY) 11: 92–94.

6. Lotka AJ (1922) The Stability of the Normal Age Distribution. Proc Natl Acad Sci U S A 8: 339–345.

7. Pollak RA (1986) A reformulation of the two-sex problem. Demography 23: 247–259.

8. Milligan BG, Stubben CJ (2007) Estimating and Analyzing Demographic Models Using the popbio Package in R. J Stat Softw 22: 1–23.
