# Supplementary figures and images for: A New Metric of Inclusive Fitness Predicts the Human Mortality Profile
Source: PLoS One. 2015 Jan 21;10(1):e0117019. doi: 10.1371/journal.pone.0117019 (PMC4301870; doi:10.1371/journal.pone.0117019)

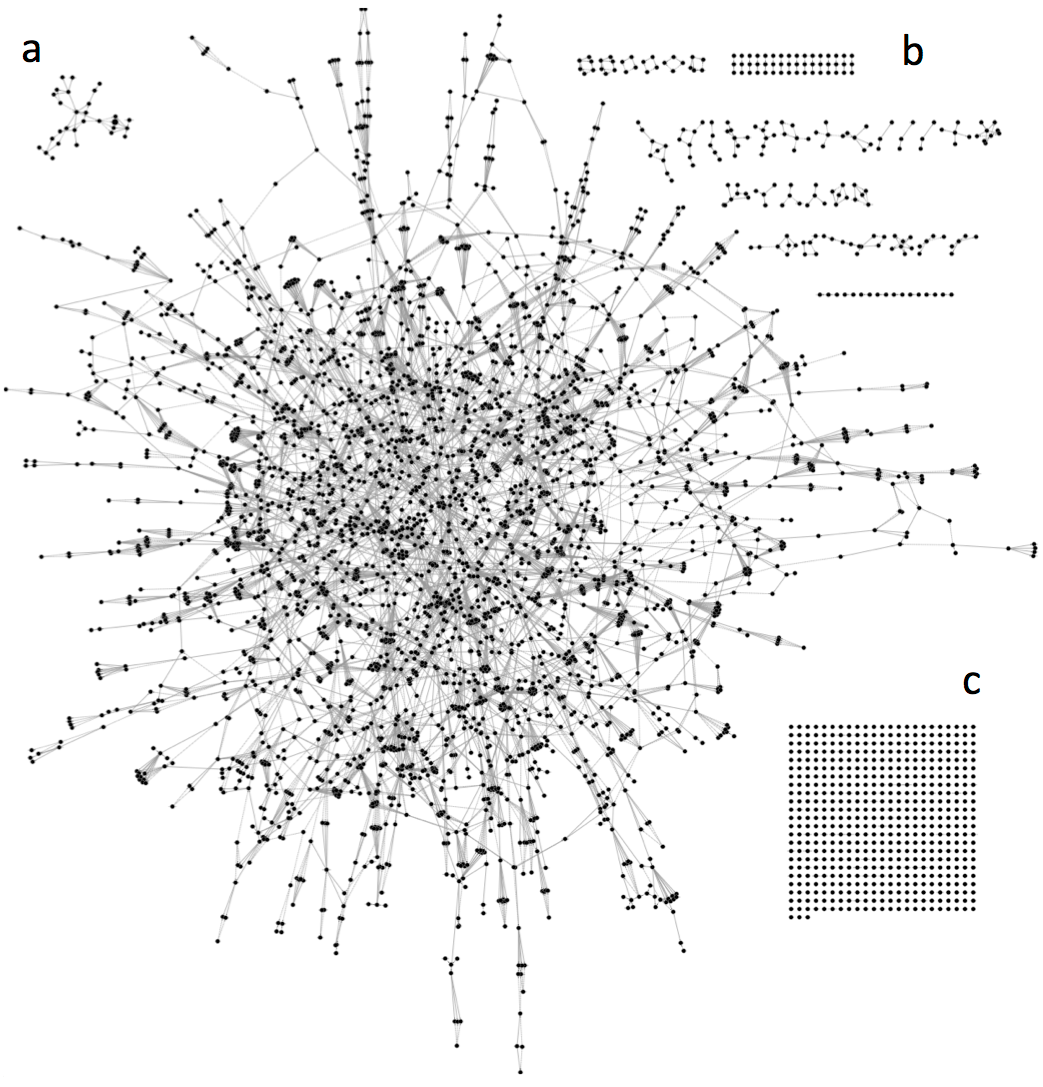

Supplement: S1 Fig — Each node is one individual, connected to its offspring by a directed edge. The population is dominated by a single kinship network (a, n = 3530), with numerous smaller unconnected kinship graphs (b, n = 266), and unrelated individuals (c, n = 532). (TIFF) [file pone.0117019.s002.tiff]

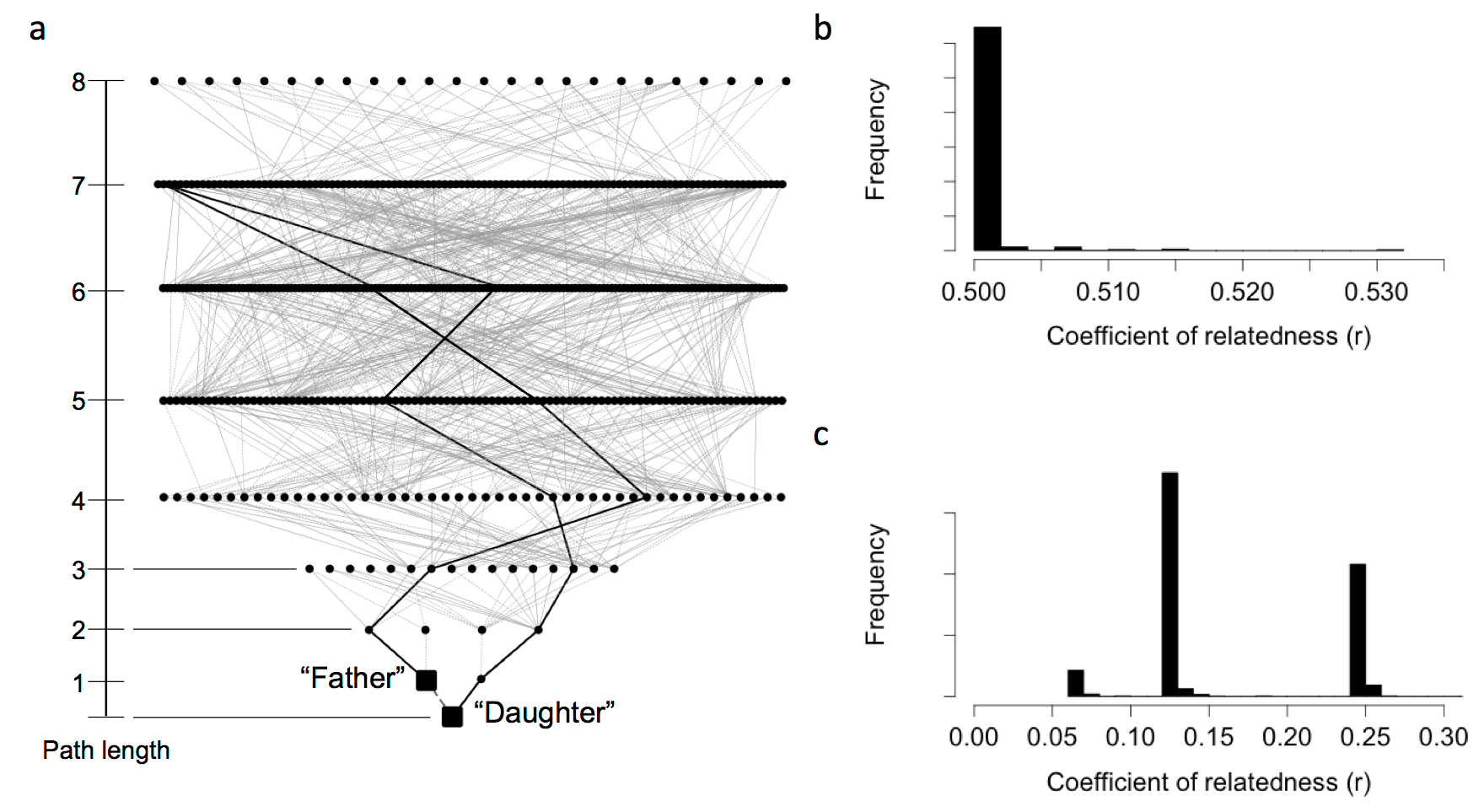

Supplement: S2 Fig — (a) Network diagram showing the diversity of paths (grey edges) connecting a “father-daughter” pair. Traditional relationships such as “father-daughter” capture information from a single genealogical path (dashed line), whereas many higher-order connections (i.e path shown in black) exist. These higher-order relationships affect coefficients of relatedness in complex patterns: for father-daughter pairs (b) deviations from the expected r = 0.5 value are relatively minor, but for higher-order relationships such as “aunt-niece” and “uncle-nephew” categorical and observed r values deviate unpredictably in complexity and magnitude (c). (TIFF) [file pone.0117019.s003.tiff]

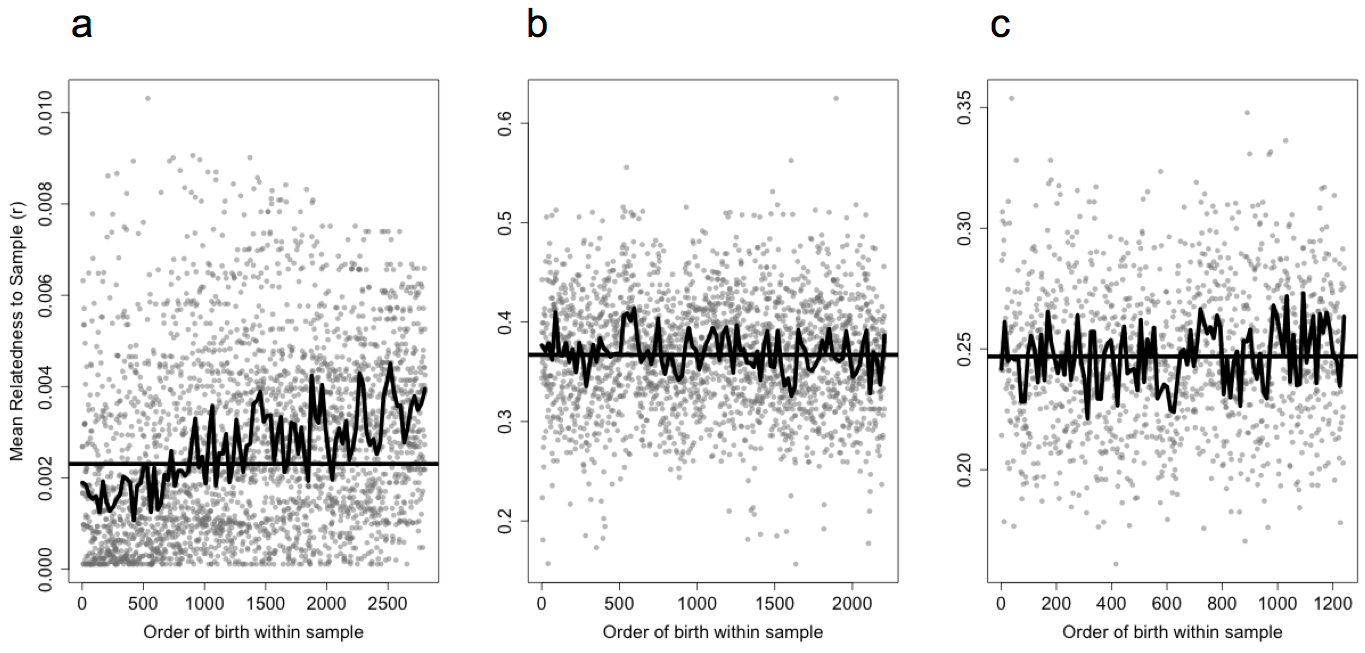

Supplement: S3 Fig — (a) The uncorrected values of r were associated with a clear bias in relatedness estimates, as individuals entering the genealogy later were on average more related to the population. The use of either parent-delimited (b) or grandparent-delimited (c) genealogies eliminated this bias by excluding higher-degree relationships from the calculation of coefficients of relatedness r. Smoothed values shown as variable black line, black horizontal line shows population mean coefficient of relatedness. (TIFF) [file pone.0117019.s004.tiff]

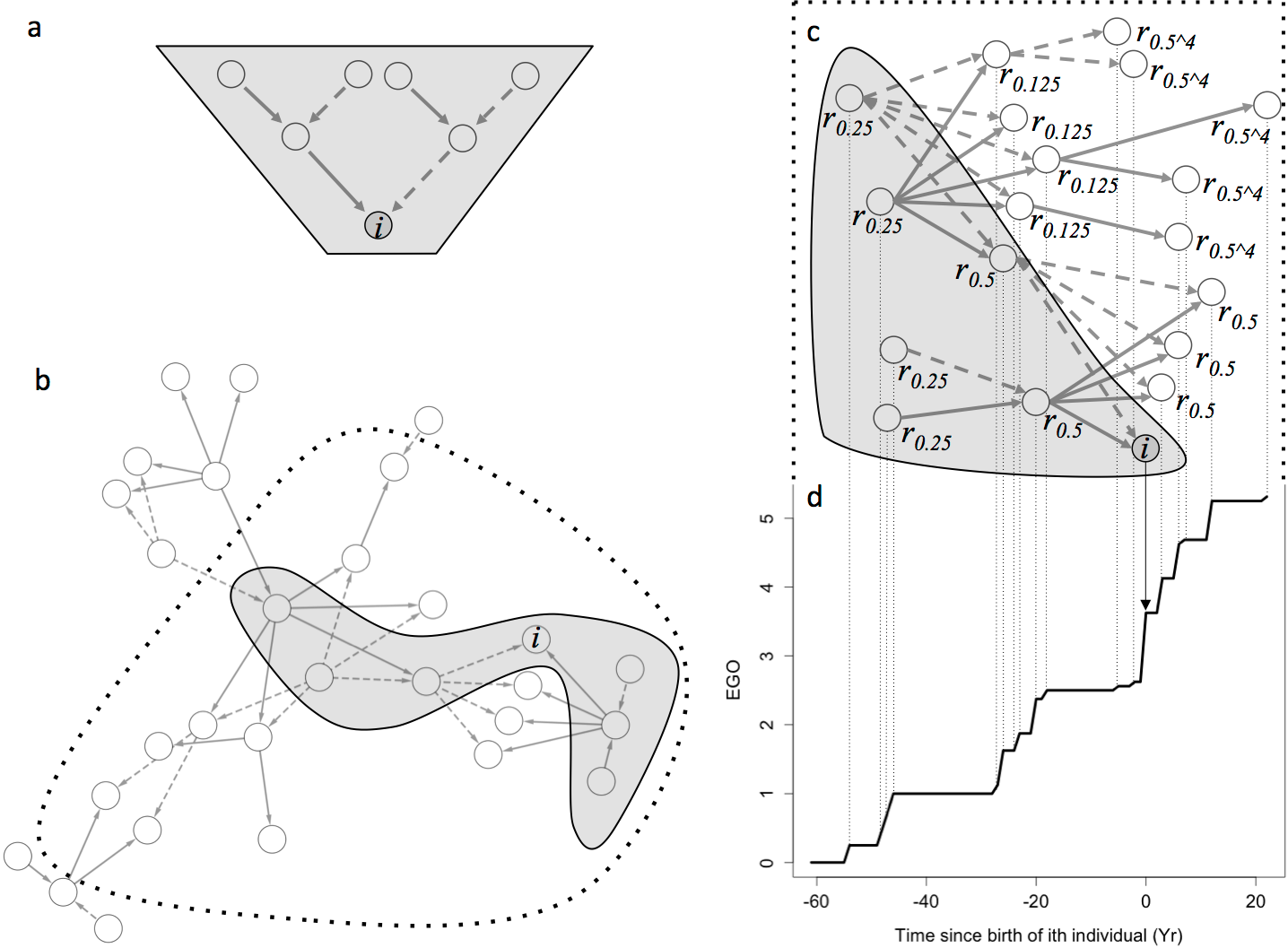

Supplement: S4 Fig — (a) A pattern-matching algorithm found every ith individual in the population with a full set of either parents or grandparents. (b) The direct descendants of all individuals in the population matching these criteria were collected (delimited by dashed line in b-c), creating a delimited sub-graph of the original genealogy. (c) We calculated the coefficient of relatedness r between the ith individual and all of their jth relatives within this sub-graph, and time-sorted the data relative to the ith individual’s date of birth. (d) We then measured the sum of r between each ith individual and their jth relatives across all periods from t = 0 to tx, where t = 0 is the earliest birth in the sub-graph and tx ranges from the birth of the ith individual to the last population census. This gave us our EGO measure of inclusive fitness over time. (TIFF) [file pone.0117019.s005.tiff]

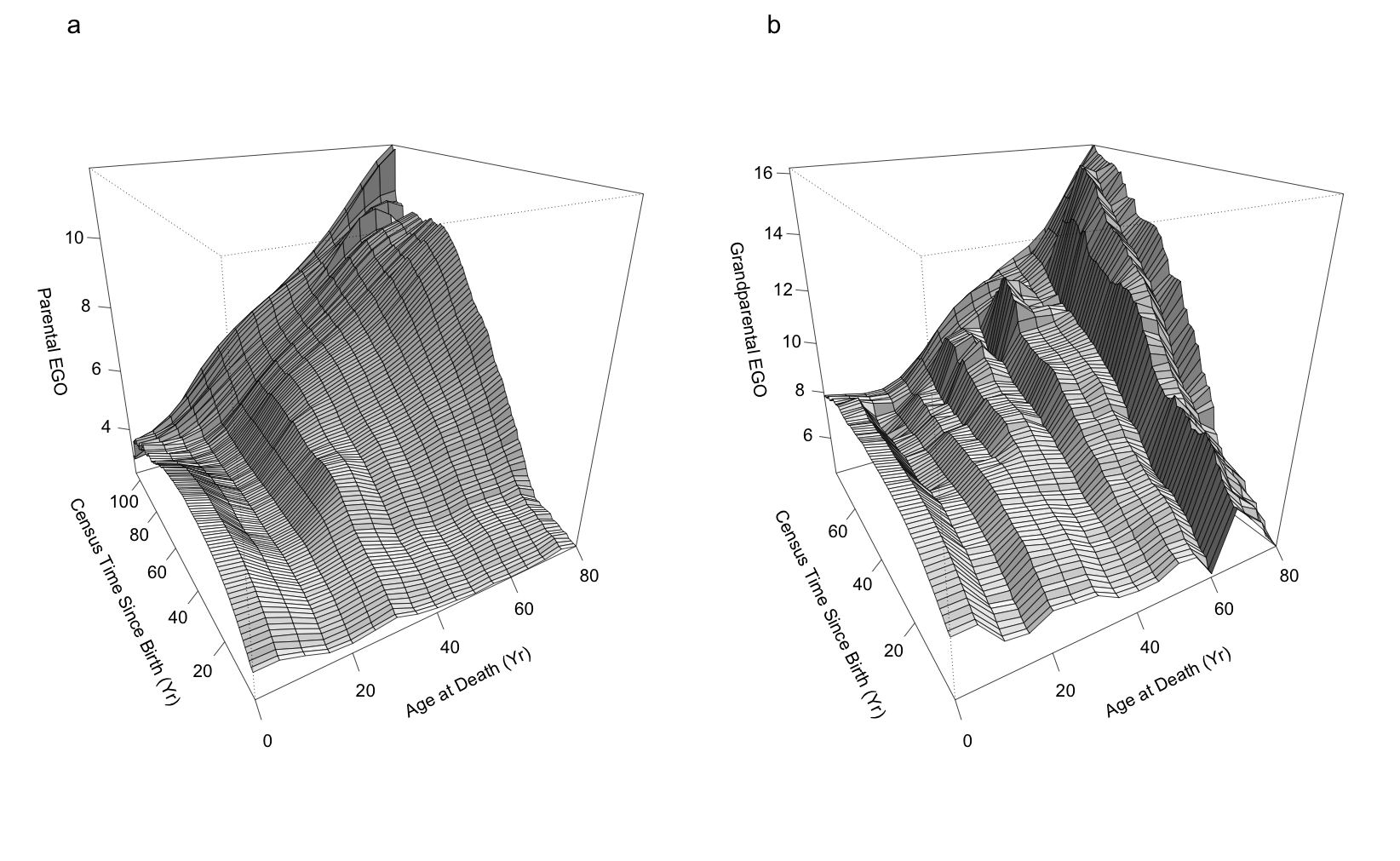

Supplement: S5 Fig — At birth, EGO is not significantly correlated with the age-specific probability of mortality (p>0.01). As time since birth increases to t > 30, both parental (a) and grandparental EGO (b) begin to closely approximate the age-specific probability of mortality (p < 0.01; r > 0.8), despite considerable reductions in sample size. (TIFF) [file pone.0117019.s006.tiff]
